# Impacts of Environmental Distractions and Interruptions on Unsupervised Digital Cognitive Assessments in Older Adults: Cognitive Ecological Momentary Assessment Study

**DOI:** 10.2196/71578

**Published:** 2025-09-08

**Authors:** Matthew Welhaf, Hannah Wilks, Andrew J Aschenbrenner, Samhita Katteri, John C Morris, Jason J Hassenstab

**Affiliations:** 1 Department of Neurology, School of Medicine, Washington University in St. Louis, 660 South Euclid Avenue, St Louis, MO, 63130, United States, 1 9548065162; 2 Department of Psychological & Brain Sciences, Washington University in St. Louis, St Louis, MO, United States

**Keywords:** ecological momentary assessment, environmental factors, cognition, Alzheimer disease, smartphone-based testing, mobile phone

## Abstract

**Background:**

Unsupervised cognitive assessments are becoming commonly used in studies of aging and neurodegenerative diseases. As assessments are completed in everyday environments and without a proctor, there are concerns about how common distractions may impact performance and whether these distractions may differentially impact those experiencing the earliest symptoms of dementia.

**Objective:**

We examined the impact of self-reported interruptions, testing location, and social context during testing on remote cognitive assessments in older adults.

**Methods:**

Participants from the Ambulatory Research in Cognition smartphone study were classified as cognitively normal (n=380) or as having very mild dementia (n=37). Participants completed daily tests of processing speed, working memory, and associative memory. At each assessment, participants were asked for their current location and social surroundings, which was used to quantify whether participants were either at home (or not) and by themselves (or not). After each assessment session, participants were asked if they experienced any interruptions. Mixed-effect modeling tested the interactions between location, social context, and clinical status. Additional analyses were conducted by removing sessions where participants reported that they were interrupted at any point during the testing period.

**Results:**

Across all participants, momentary effects of environmental distractions were minimal. Specifically, when tests were completed in the presence of others, participants exhibited slightly increased variability in processing speed (*P*=.04). However, these momentary effects of environmental distractions were dependent upon cognitive status (*P*=.009). Cognitively normal older adults had better visuospatial working memory performance when they completed tests at home compared to when they completed tests away from home (*P*=.001). However, older adults with very mild dementia showed no effect of testing location on the same task (*P*=.36). Conversely, cognitively normal older adults did not differ in their processing speed at either testing location (*P*=.88). Older adults with very mild dementia were slightly faster when not at home (*P*=.04). Social context only impacted variability in processing speed for participants with very mild dementia (*P*=.04). When considering tests completed in the most distracting environments (away from home and in the presence of others), those with very mild dementia showed larger differences only on the visuospatial working memory measure. Additional analyses demonstrated that after removing sessions in which participants self-reported experiencing an interruption (1194/9633, 12.4% of all assessments), these small effects of environmental distractions on cognition remained, but were more apparent in those with very mild dementia.

**Conclusions:**

Social context and location of unsupervised remote cognitive testing have small impacts on performance, but these impacts were not consistent across cognitive domains and were mostly limited to participants demonstrating the earliest symptoms of dementia. Remote cognitive testing provides valid and reliable data in older adults, but care should be taken to allow participants to report distractions that may occur during testing.

## Introduction

It is becoming increasingly common to use remote cognitive assessments to track fluctuations in cognitive performance in older adults [1-6]. These tools are especially useful for measuring subtle fluctuations in cognition during the preclinical stage of Alzheimer disease (AD) [3,4,7-15]. However, because remote testing is typically done in a participant’s home, usually without the supervision of a test proctor or clinician, it is hard to know the extent to which the surrounding environment is controlled and distraction-free. Distractions occur in approximately 7%‐10% of at-home cognitive testing sessions [8,16]. Such distractions can lead to worse performance on remote cognitive assessments because they might pull attention away from the task, which could lead to participants making more mistakes and having slower and more variable reaction times (RTs).

Ecological momentary assessment (EMA) is a powerful sampling method that leverages the variability in environmental factors to assess psychological processes in participants’ everyday lives. EMA has been used to provide a more naturalistic and sensitive assessment of cognition (and other psychological factors) [2,17] and may provide a more reliable sampling of a participant’s cognitive performance that matches their true cognitive ability [15,17]. EMA studies often consider external factors that might impact cognitive performance, such as time of day [18], mood or affect [19], current and recent stressors [20,21], everyday activities such as chores [7,22], and recent physical activities [23]. However, because EMA studies collect data during participants’ everyday lives, it is possible that distractions introduced by the environment are more prominent and impact performance more when compared to in-clinic or even remotely proctored assessments. A recent review [24] suggests that testing location or social context can have some meaningful impact on performance on remote cognitive tests (both proctored and unsupervised), but the literature has been limited to assessments in a single age group or in a single cognitive domain, so the generalizability of the findings is unknown. Supporting this claim, a recent empirical study found that during remote cognitive assessments, participants who provided data for normative purposes performed better on measures of processing speed when they completed tests at home and also performed better on measures of recognition and working memory when they completed tests alone [25].

Depending on study goals, environmental distractions may be considered confounds that need to be controlled for in EMA studies of cognition. In studies of aging and neurodegenerative disease, researchers are interested in capturing subtle changes in cognition related to changes in neurological functioning, so understanding the effects of different sources of distraction is critical. Recent work has addressed the potential confounding effect of contextual factors such as environmental and social distractions on remote cognitive performance among cognitively impaired and cognitively unimpaired older adults [26]. Cerino et al [26] reported that there were small effects of environmental and social distractions that were evident on measures of visual short-term memory binding and spatial working memory. Although the effects were not large, they were still significant across all participants, even those with mild cognitive impairment (MCI), suggesting that environmental and social distractions can affect cognition beyond disease-related impairments.

It is important to consider the extent to which those showing the very earliest symptoms of dementia are affected by these distractors compared to cognitively normal individuals. That is, subtle deficits may become more apparent for those experiencing the very earliest symptoms of dementia when tests are completed “in the wild,” away from home, or when around others. The goal of this study was to examine the impact of environmental and social distractions during remote cognitive testing in a well-characterized cohort of older adults enrolled in a study of normal aging and dementia. We hypothesized that symptomatic participants would be more impacted by environmental distractions compared to cognitively normal participants, especially when tests were taken in the least optimal settings (ie, both away from home and in the presence of others). Consistent with previous work [26], we operationalized testing location as assessments completed at home (versus not), and social environment as assessments that were completed alone versus in the presence of others.

## Methods

### Participants

Participants were recruited from studies of aging and dementia at the Knight Alzheimer Disease Research Center at Washington University School of Medicine in St. Louis. The Ambulatory Research in Cognition (ARC) study began in March of 2020. ARC is a custom-built smartphone app that assesses cognition with repeated brief assessments up to 4 times per day for one week [13]. Participants were invited to enroll after completing their annual cognitive and clinical assessment and began ARC testing shortly after enrolling. As data collection is still ongoing, we used a data freeze from June 2024 for the current analyses.

For the current analyses, participants were included if they (1) completed at least 10 sessions during their baseline ARC testing to ensure adequate engagement in the study and enough observations in different environments for comparisons and (2) had a clinical assessment (see below) within a year of starting their ARC testing to ensure an accurate classification of cognitive status.

### Clinical Assessments

Clinical status was assessed using the Clinical Dementia Rating (CDR) [27]. The CDR rates cognitive and functional performance on a 5-point scale across 6 domains (memory, orientation, judgment and problem solving, community affairs, home and hobbies, and personal care). Participants and a collateral source (a close family member or friend) both underwent semistructured interviews to determine CDR scores, with CDRs of 0 indicating cognitive normality, 0.5 indicating very mild dementia, 1 indicating mild dementia, 2 indicating moderate dementia, and 3 indicating severe dementia. For this study, we grouped participants as either cognitively normal (CDR 0) or diagnosed with very mild dementia (CDR 0.5). The parent study was designed to characterize the transition from cognitive normality to the very first signs of clinical symptoms of AD; therefore, most participants were cognitively normal (CDR 0), with the final sample consisting of 385 cognitively normal participants and 37 participants with a CDR of 0.5. Of the 37 participants with CDR 0.5, a total of 26 (70%) were clinically diagnosed with AD, and the rest with a dementia of different etiology, including Parkinson disease, Lewy body dementia, or uncertain etiology. CDR assessments were completed 7.64 (SD 7.78) weeks from their ARC assessment.

### Smartphone Assessment

**Figure 1.**
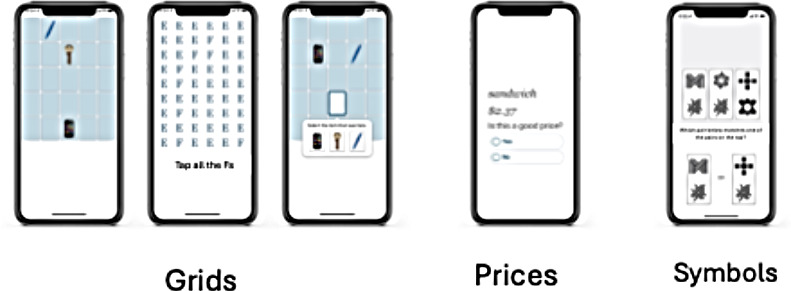
Examples of grids, prices, and symbols tests from Ambulatory Research in Cognition (ARC).

Methods for the procedures of ARC are detailed elsewhere, so are only briefly described here (for full details on the ARC platform, see studies by Nicosia et al [13] and Wilks et al [18]). Participants completed all ARC assessments on a smartphone (either their own or a study-provided device). ARC assessments were sent to participants using the native iOS (Apple Inc) or Android (Google LLC) notification systems pseudorandomly. Participants were instructed to complete the assessment as soon as possible within a 2-hour window. Participants completed 3 cognitive tasks (described below) up to 4 times per day over the course of a week. Figure 1 provides screenshots of each task presented on ARC.

### Symbols

During this processing speed task, participants were randomly shown 3 pairs of abstract shapes and were asked to select which of 2 possible responses matched 1 of the 3 targets [17]. At each assessment, participants completed 12 trials lasting approximately 20‐60 seconds. Consistent with our previous work [13,18,28], we analyzed symbols’ performance in 2 ways. The first was the median RT of correct trials, which we use to capture differences in processing speed. We also calculated a measure of RT variability, the coefficient of variation (CoV). For both median RT and RT CoV, higher scores reflect poorer performance (ie, slower responding or greater variability).

### Prices

This associate memory task presented subjects with 2 phases: a learning phase and a recognition phase. During learning, participants were instructed to learn and remember 10 item-price pairs that were presented on screen for 3 seconds per pair. The items were everyday shopping items (eg, foods or household supplies), and prices were randomly assigned 3-digit prices. During the recognition phase, participants were presented with an item they encountered during learning and 2 prices and were asked to choose which price was originally paired with the item. Each administration of this test requires approximately 60 seconds. The primary dependent measure for this task was the error rate during recognition, such that higher scores indicated poorer recognition.

### Grids

In this spatial working memory task, participants were presented with images of three everyday objects (key, smartphone, and pen) on a 5x5 grid. Participants were told to remember the location of each item on the grid. After the encoding phase, participants were given a distractor task (locating Fs among a grid of Es). Following the distractor task, participants were asked to recall the location of each item on a blank grid. Participants completed 2 trials at each session (total time 30‐40 s). The primary dependent measure for this task was the Euclidean distance for each item. Higher scores indicated that participants placed the items further away from the initially encoded location and suggested worse performance [17].

### Current Environment and Social Context

At each ARC session, participants answered several questions about their experiences or situations at the time of the notification. The first question, which we used to operationalize testing location, asked, “Where are you right now?*”* Participants selected one of the following response options: my home, my work, school, another person’s home, vehicle, outside, or other. We categorized participants’ responses into either home (if they answered “my home”) or away (all other responses).

The second question, which we used to operationalize social context, asked, “Who is with you right now?” Participants could select all that apply from the following options: nobody, partner or spouse, family, friends, coworkers, pet, and other. As above, we categorized participants’ responses into either testing alone (if they answered “nobody”) or not alone (all other responses). These environment and social context categorizations were consistent with a previous study [26]. As displayed in Table 1, most participants completed their tests at home and alone, with few reports in other contexts. As there was little variation within each category, we chose not to treat these as separate variables in analyses. Additionally, there is some debate in the field of EMA on whether “pets” should be counted as alone or not alone. We considered responses of “pets” as not alone, as it is likely that pets might also be a source of distraction for participants during remote cognitive assessments (eg, a dog barking at a passing car might be a salient distraction). The results were nearly identical when including “pet” responses as alone.

**Table 1.** Demographics of sample by CDR^a^ status. Gender and race were self-reported.

Demographic variables	CDR 0 (n=380)	CDR=0.5 (n=37)	Statistic	*P* value
Age (years), mean (SD)	75.42 (5.93)	76.57 (6.32)	1.07 (42.76)^b^	.29
Gender, n (%)	226 (56.8)	14 (37.8)	5.17 (1)^c^	.02
Education (years), mean (SD)	16.49 (2.42)	16.08 (2.19)	1.08(44.90)^b^	.28
Race, n (%)				
White	322 (84.7)	33 (89.1)		
Black or African American	61 (15.1)	4 (10.8)		
Other	2 (< 1)	0 (0)		
APOE ε4^d^ status, n (%)			8.80 (1)^c^	.003
ε4 carrier	129 (33.5)	22 (59.5)		
ε4 noncarrier	256 (66.5)	15 (40.5)		
Sessions complete, mean (SD)	23.22 (4.29)	21.92 (5.40)	1.42 (40.49)^b^	.16
Prices error rate, mean (SD)	0.29 (0.09)	0.37 (0.08)	–5.40 (45.43)^b^	<.001
Grids Euclidean distance, mean (SD)	0.74 (0.26)	0.95 (0.26)	–4.72 (43.23)^b^	<.001
Symbols median RT^e^, mean (SD)	3.25 (1.08)	3.98 (1.27)	–3.39 (41.09)^b^	.002
Symbols RT CoV^f^, mean (SD)	0.34 (0.08)	0.39 (0.10)	–2.91 (40.21)^b^	.006
Proportion completed at home, mean (SD)	0.80 (0.19)	0.86 (0.15)	–2.19 (48.76)^b^	.03
Proportion completed alone, mean (SD)	0.49 (0.32)	0.43 (0.32)	1.16 (42.93)^b^	.25

a CDR: Clinical Dementia Rating.

b *t (df)*

c Chi-square (*df*)

d *APOE* ε4*: *apolipoprotein ε4.

e RT: reaction time.

f CoV: coefficient of variation.

### Self-Reported Interruptions

Upon completion of each session, participants were asked if they experienced any interruptions during the testing period. This item was used in additional analyses (described below) to account for sessions where participants were acutely aware of potential distractions.

### Analytic Plan

Our primary analyses addressed how the environmental factors affect the relationship between CDR status and performance in remote unsupervised testing. In each linear mixed effects model, the outcome measure was either symbols median RT, symbols CoV, prices error rate, or grids Euclidean distance. We included fixed effects of age at ARC testing, years of education, self-reported gender (male or female), and number of completed sessions (to account for practice effects). We also included a fixed effect term for the proportion of sessions completed at home and the proportion of sessions completed alone to account for between-person differences in the testing location or context. That is, some people might complete more ARC assessments at home, and some might complete more assessments alone than others. To account for within-person variance, we included a term for the reported location (ie, home or not home; coded 0 and 1, respectively) and social context (ie, alone or not alone; coded 0 and 1, respectively) at each ARC session. We included all 2-way interactions between CDR status, within-person location, and within-person social context, and the higher-order 3-way interaction as fixed effects. These terms allowed us to test the differential impact that environmental distractions had on both cognitively normal older adults and those with very mild dementia. All models included a random intercept for the participant.

In our second set of analyses, we removed sessions where, upon completion of each assessment session, participants reported being interrupted. We tested the hypothesis that if participants were aware of their distractions and reported them, then removing these sessions should greatly reduce or eliminate any effects of location or social context on cognitive performance. Conversely, if location and social context effects are still apparent, then we would demonstrate that environmental distractions impact remote cognitive performance beyond instances in which participants are overtly interrupted.

All mixed-effect models were conducted using R software (version 4.4.0; R Foundation for Statistical Computing) including the *lmer* package [29] and *P* values for mixed-effect models were estimated using the *lmerTest* [30] package. Follow-up comparisons were conducted using *emmeans* [31].

### Ethical Considerations

All participants provided informed consent, and all procedures were approved by the Human Research Protections Office at Washington University in St. Louis (IRB# 201907164), and the research was conducted in accordance with the Helsinki Declaration. Participants’ data were fully anonymized during the data collection process, and researchers cannot access any identifiable participant information without requesting access to the data via a formal data request to the Knight Alzheimer Disease Research Center Administrative Core. As described in Nicosia et al [13], participants were paid US $0.50 per completed assessment session. Participants also received bonus payments for completing all 4 sessions on any given day (US $1.00 per occurrence, max of US $7.00), completing at least 2 assessments per day for 7 days (US $6.00), and completing at least 21 assessments over 7 days (US $5.00). The maximum compensation possible for one 7-day assessment visit was US $32.00.

## Results

### Participant Demographics

The final sample consisted of 417 participants (see Table 1). Table 2 provides demographic and ARC task information on the full sample, grouped by clinical status. Participants were highly educated, most self-identified their race as White, and were a majority of females. Adherence was high with participants completing, on average, 23.11 (SD 4.41) out of a possible 28 sessions (82.54% adherence).

**Table 2.** Mean (SD) and percentage of testing locations and social contexts reported by participants for both cognitively normal and those with very mild dementia across the week of testing (maximum of 28 sessions).

Question	CDR^a^ 0 (n=380)		CDR=0.5 (n=37)	
Where are you right now?	Mean (SD)	Total observations, n (%)	Mean (SD)	Total observations, n (%)
My home	18.77 (5.57)	7131 (81)	19.03 (6.09)	704 (86)
My work	0.50 (1.67)	190 (2)	0.00 (0.00)	0 (0)
School	0.02 (0.17)	9 (<1)	0.00 (0.00)	0 (0)
Another person’s home	0.82 (2.16)	311 (4)	0.46 (1.30)	17 (2)
Vehicle	1.20 (1.54)	455 (5)	1.19 (1.37)	44 (6)
Outside	0.60 (1.15)	229 (3)	0.35 (0.63)	13 (2)
Other	1.25 (2.12)	476 (5)	0.89 (2.11)	33 (4)
Who is with you right now?				
Nobody	11.33 (7.69)	4306 (49)	9.38 (7.65)	347 (43)
Partner or spouse	5.42 (6.57)	2060 (23)	7.62 (7.55)	282 (34)
Family	1.62 (3.59)	616 (7)	1.24 (3.68)	46 (6)
Friends	0.61 (1.30)	232 (3)	0.16 (0.37)	6 (1)
Coworkers	0.12 (0.64)	44 (<1)	0.03 (0.16)	1 (<1)
Pet	2.01 (4.94)	764 (8)	1.35 (4.13)	50 (7)
Other	0.49 (1.48)	188 (2)	0.22 (0.63)	8 (1)
Multiple	1.55 (3.70)	590 (7)	1.92 (3.85)	71 (9)

a CDR: Clinical Dementia Rating.

As expected, participants with very mild dementia performed worse on the ARC assessments when aggregated across the week of testing. Further, there was a small difference in the proportion of tests completed at home, such that individuals with very mild dementia completed a greater proportion of tests at home compared to cognitively normal participants (n=704, 86% vs n=7131, 81%, respectively). There was no difference between cognitively normal participants and those with very mild dementia in the proportion of tests completed alone. Table 1 provides more detailed information regarding the response frequencies to both the location and social surrounding questions for both cognitively normal participants and those with very mild dementia.

### Effects of Testing Location and Social Context

Consistent with our other publications in this sample [13,18,28,32], older age at the ARC assessment predicted worse performance on all ARC measures (Table S1 in Multimedia Appendix 1). Male participants made more errors than females on the prices test but were slightly better at the grids test. More years of education were also associated with better performance on prices and grids (but not symbols). Participants who completed more sessions performed better on grids and had faster processing speed and lower variability over time on symbols indicating evidence of practice effects on these tasks.

As seen in Table 3, neither the proportion of tests completed at home (*ß*’s=–0.131 to 0.072, *P*’s>.31) nor the proportion of tests completed alone (*ß*’s=–0.309 to 0.020, *P*’s>.06) was related to performance on any of the ARC tasks. When considering the session-level effects of testing location, there were no significant effects of testing location on ARC performance. Participants showed similar performance on all ARC measures regardless of testing location: prices (*M*_home_ 0.327, SE 0.007 vs *M*_not home_ 0.354, SE 0.011, *t*_9399_=–2.773, *P*=.006, Cohen *d*=–0.06, 95% CI –0.10 to –0.02), grids (*M*_home_ 0.816, SE 0.02 vs *M*_not home_ 0.813, SE 0.03, *t*_9217_=–0.118, *P*=.91, *d* < –0.01, 95% CI –0.04 to 0.04), symbols processing speed (*M*_home_ 3.52, SE 0.09 vs *M*_not home_ 3.43, SE 0.10, *t*_9399_=1.925, *P*=.054, *d*=0.04, 95% CI 0.00 to 0.08), symbols variability in processing speed (*M*_home_ 0.361, SE 0.007 vs *M*_not home_ 0.372, SE 0.01, *t*_9366_=–1.362, *P*=.17, *d*=–0.03, 95% CI –0.07 to 0.01). The main effect on prices performance is significant when calculating the simple effect; however, caution should be used when interpreting this effect, as it is likely driven by the interaction between testing location and social context.

**Table 3.** Mixed effect models of the interaction between CDR^a^ status, testing location, social context on ARC^b^ performance (N=417). Exact *P* values are presented in the text. Models adjusted for covariates, including age, years of education, self-reported gender, and number of completed sessions. Full model output is provided Table S1 in Multimedia Appendix 1.

Predictors	Prices error rate, estimate (SE)	Grids Euclidean distance, estimate (SE)	Symbols median RT^c^, estimate (SE)	Symbols RT CoV^d^, estimate (SE)
Home proportion^e^	–0.015 (0.024)	0.072 (0.072)	–0.131 (0.286)	–0.001 (0.022)
Alone proportion^f^	0.001 (0.014)	0.020 (0.041)	–0.309 (0.163)	0.016 (0.013)
Home^g^	0.004 (0.007)	0.017 (0.019)	–0.032 (0.033)	0.003 (0.006)
Alone^h^	0.006 (0.005)	0.011 (0.012)	–0.022 (0.021)	0.008^i^ (0.004)
CDR status x home	0.046 (0.033)	–0.218^i^(0.087)	–0.384^i^(0.152)	0.021 (0.027)
CDR status x alone	0.004 (0.015)	0.038 (0.040)	–0.049 (0.071)	0.032^j^ (0.012)
Home or not x alone	0.024^j^ (0.009)	0.048^i^ (0.024)	0.070 (0.042)	0.002 (0.008)
CDR status x home x alone	–0.045 (0.039)	0.260^i^ (0.102)	0.401^i^ (0.178)	–0.015 (0.032)

a CDR: Clinical Dementia Rating.

b ARC: Ambulatory Research in Cognition.

c RT: reaction time.

d CoV: coefficient of variation.

e Home proportion: proportion of sessions completed at home.

f Alone proportion: proportion of sessions completed alone.

g Home: session completed at home (coded 0) or away from home (coded 1).

h Alone: session completed alone (coded 0) or in the presence of others (coded 1).

i *P<.05.

j **P<.01.

This was also largely the case for session-level effects of social context. Participants showed similar performance, regardless of social context, on prices (*M*_alone_ 0.336, SE 0.01 vs *M*_not alone_ 0.345, SE 0.009, *t*_9504_=–0.871, *P*=.38, *d*=–0.02, 95% CI –0.06 to 0.02]), grids (*M*_alone_ 0.755, SE 0.03 vs *M*_not alone_ 0.874, SE 0.03, *t*_9333_=–4.398, *P*<.001, *d*=–0.09, 95% CI –0.13 to –0.05), and symbols processing speed (*M*_alone_ 3.43, SE 0.10 vs *M*_not alone_ 3.52, SE 0.09, *t*_9223_=–1.878, *P*=.06, *d*=–0.04, 95% CI –0.08 to 0.00). Again, this seemingly large effect is likely driven by higher-order interactions, and so caution should be used when interpreting this effect.

However, there was a significant effect on the variability measure from the symbols task, such that when participants completed sessions in the presence of others (mean 0.377, SE 0.008), they demonstrated increased variability in RTs on the symbols task compared to when they completed sessions alone (mean 0.356, SE 0.009; *t*_9469_=–2.563, *P*=.04, *d*=–0.05, 95% CI –0.09 to –0.01).

Performance on 3 ARC measures was significantly impacted by both CDR status and testing location or social context (ie, significant 2-way interactions; Table 3). For grid performance, cognitively normal older adults performed better when they completed tests at home compared to when they completed tests away from home (*M*_home_ 0.714, SE 0.01 vs *M*_not home_ 0.755, SE 0.02, *t*_9036_=–3.317, *P*=.001). However, older adults with very mild dementia did not differ in their grids performance in different testing environments (*M*_home_ 0.918, SE 0.04 vs *M*_not home_ 0.871, SE 0.06, *t*_9036_=–3.317, *P*=.001). For symbols processing speed, cognitively normal older adults did not differ in either testing location (*M*_home_ 3.22, SE 0.05 vs *M*_not home_ 3.23, SE 0.06, *t*_9152_=–0.157, *P*=.88). However, older adults with very mild dementia showed a significant, albeit small, difference in processing speed based on their testing location (*M*_home_ 3.81, SE 0.17) vs *M*_not home_ 3.63, SE 0.19, *t*_9254_=0.925, *P*=.36).

As for social context, there was a significant interaction with CDR status on RT variability on the symbols task. When cognitively normal participants completed the test alone, they were significantly less variable compared to when they completed the tests in the presence of others (*M*_alone_ 0.337, SE 0.004 vs *M*_not alone_ 0.346, SE 0.05, *t*_9261_=–4.650, *P*=.02). This effect was larger for participants with very mild dementia who completed tests (*M*_alone_ 0.375, SE 0.02 vs *M*_not alone_ 0.409, SE 0.01, *t*_9510_=–2.085, *P*=.04).

Finally, across all participants, there was evidence that the combination of taking tests away from home and in the presence of others significantly impacted performance on prices and grids. Specifically, when tests were taken in the most suboptimal locations (ie, away from home and in the presence of others), participants, on average, made significantly more errors on prices (mean 0.359, SE 0.01) compared to when they completed the test in “optimal” contexts (ie, at home and alone; mean 0.323, SE 0.008; *t*_9535_=–3.218, *P*=.001). This was also evident for grids as participants were, on average, worse when completing both away from home and in the presence of others (mean 0.917, SE 0.03) compared to when they completed tests at home and alone (mean 0.801, SE 0.02; *t*_9170_=2.099, *P*=.04).

**Figure 2.**
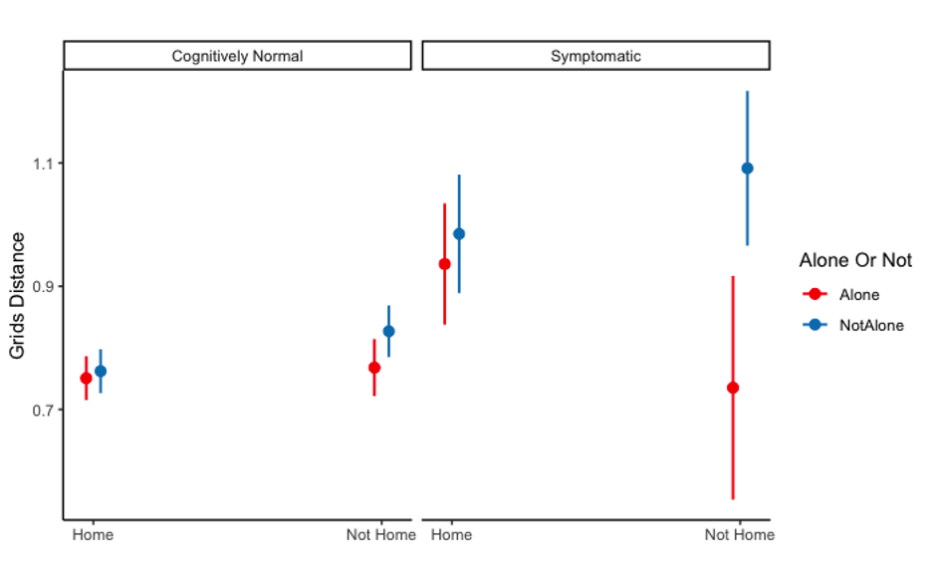
Estimated effects of testing location and social context on grids for cognitively normal (CDR 0; n=380) and symptomatic (CDR 0.5; n=37) participants. Error bars represent the 95% CI. CDR: Clinical Dementia Rating.

**Figure 3.**
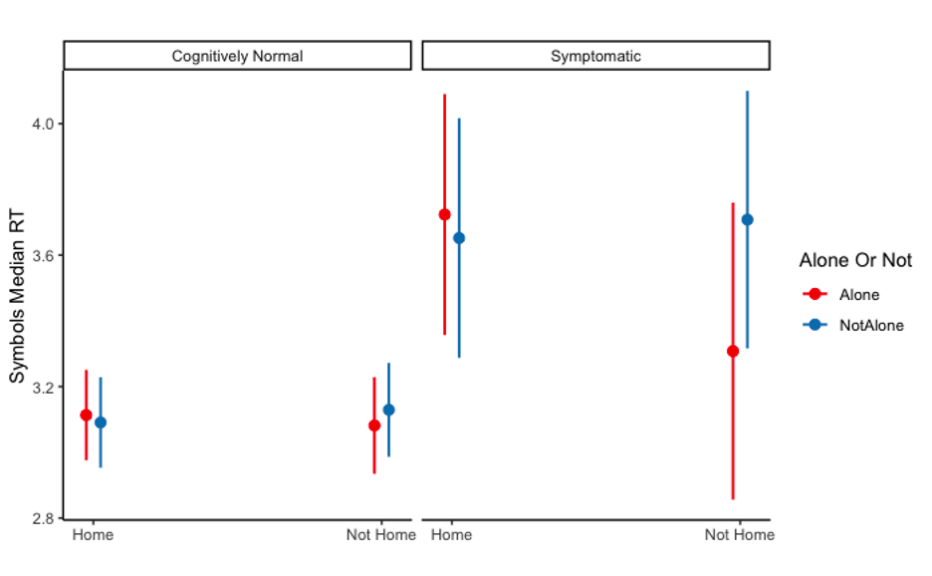
Estimated effects of testing location and social context on symbol median RT for cognitively normal (CDR 0; n=380) and symptomatic (CDR 0.5; n=37) participants. Error bars represent the 95% CI. CDR: Clinical Dementia Rating; RT: reaction time.

These lower-order interactions were qualified by 3-way interactions on grids (Figure 2) and symbols median RT (Figure 3). Decomposing the interaction suggested that when those with very mild dementia completed tests both away from home and in the presence of others, they had an average grids score of 1.049 (SE 0.06), which was significantly higher than when they completed tests at home and alone (mean 0.894, SE 0.05; *t*_9399_=–2.754, *P*=.006, *d*=–0.05, 95% CI –0.09 to –0.01]). For processing speed, however, the difference between the optimal (mean 3.85 s, SE 0.18) and suboptimal (mean 3.83 s, SE 0.19) environments was not statistically significant (*P*=.88).

### Removing Interrupted Sessions

Interruptions were reported on 1194 of 9633 completed sessions (approximately 12% of all sessions). Removing these sessions resulted in an overall similar pattern of effects (Table 4), albeit an attenuated effect. The previously described 3-way interactions between CDR status, testing location, and social context were still evident on the grids and symbols median RT. Thus, even after removing sessions during which participants reported experiencing an overt interruption, the effects of environmental distractions were still apparent, especially for those with very mild dementia.

**Table 4.** Mixed effect models of the interaction between CDR^a^ status, testing location, and social context on ARC^b^ performance after excluding sessions that were interrupted (N=417). Models adjusted for covariates, including age, years of education, self-reported gender, and number of completed sessions. Full model output is provided in Table S2 of Multimedia Appendix 1.

Predictors	Prices error rate, estimate (SE)	Grids Euclidean distance, estimate (SE)	Symbols median RT^c^, estimate (SE)	Symbols RT CoV^d^, estimate (SE)
Home proportion^e^	–0.015 (0.024)	0.051 (0.072)	–0.087 (0.269)	0.001 (0.020)
Alone proportion^f^	0.005 (0.014)	0.019 (0.042)	–0.286 (0.152)	0.022 (0.012)
Home^g^	0.003 (0.008)	0.009 (0.020)	–0.062 (0.034)	–0.000 (0.006)
Alone^h^	0.006 (0.005)	0.008 (0.013)	–0.037 (0.022)	0.009^i^(0.004)
CDR status x home	0.055 (0.033)	–0.243^j^ (0.086)	–0.404^j^ (0.148)	0.025 (0.026)
CDR status x alone	0.002 (0.016)	0.029 (0.041)	–0.061 (0.072)	0.034^j^ (0.012)
Home or not x alone	0.022^i^(0.010)	0.034 (0.026)	0.094^i^ (0.045)	0.001 (0.008)
CDR status x home x alone	–0.057 (0.040)	0.287^j^ (0.102)	0.380^i^ (0.176)	–0.030 (0.031)

a CDR: Clinical Dementia Rating.

b ARC: Ambulatory Research in Cognition.

c RT: reaction time.

d CoV: coefficient of variation.

e Home proportion: proportion of sessions completed at home.

f Alone proportion: proportion of sessions completed alone.

g Home: session completed at home (coded 0) or away from home (coded 1).

h Alone: session completed alone (coded 0) or in the presence of others (coded 1).

i *P<.05

j **P<.01

## Discussion

### Principal Findings

The overall results of this study illustrate the importance of considering environmental factors in remote studies of cognition where participants may complete assessments in different locations and social contexts. The effect sizes of distractions on cognition were generally small in magnitude and likely would not invalidate remote testing, but distractions may be most important to consider in populations where cognitive impairment is evident.

Across participants, in-the-moment effects of testing location and social context were evident on measures of associative memory and visual working memory but not processing speed. When participants completed tests in the most distracting environments (both away from home and around others), they performed worse. However, for cognitively normal older adults, the impacts of these environmental factors were minimal, if at all apparent. On the other hand, participants with very mild dementia were the most sensitive to distractions, specifically when completing tests away from their homes and in the presence of others. These findings indicate that an inability to maintain performance in the face of distractions may be a novel indicator of very early neurodegenerative disease and emphasize the need for remote studies to carefully consider testing environments. While a strength of EMA is that it can allow for the measurement of cognition in a variety of environments and situations, the potential for distractions should at least be evaluated and, if necessary, included in statistical models. This is particularly true for studies enrolling individuals with very mild dementia symptoms.

The descriptive results from this study revealed that older adult participants engaged in remote cognitive assessments outside of their homes during 81% of assessments and in the company of others during 44% of assessments, both of which are environments that are likely to produce distractions. Our estimates of tests completed in these environments closely match that of Cerino et al [26], who found that older adults in their study reported testing at home in 78% of assessments and testing alone for 44%. Interestingly, Cerino et al [26] reported that participants with MCI completed slightly more tests at home compared to cognitively normal participants (*P*=.08). Results from our sample replicated these patterns as we found a significant difference between the frequency of tests completed at home between cognitively normal participants and those with a CDR of 0.5, indicating the presence of a very mild dementia. A CDR of 0.5 can be considered very similar to a classification of MCI [33,34]. This suggests that older adults with cognitive difficulties may spend more time at home or may avoid taking tests outside of the home, perhaps due to the impacts of environmental distractions.

Participants in our study have the option to report if they experienced a significant interruption during testing. Interrupted sessions were not uncommon for our participants, as nearly 12% of sessions were reported as having some form of interruption. Interestingly, the effects of environment, while reduced, remained significant after removing sessions where participants specifically flagged interruptions. Thus, aspects of environmental context that may not rise to the level of an overt interruption may have deleterious effects on cognition, and these effects appear to be magnified in those with dementia symptoms.

### Implications

The results of this study have several implications for EMA studies of cognition in older adults at risk for AD. First, the descriptive results showed that a large proportion of assessments are completed at home. Thus, it is possible that we are not fully sampling participants’ everyday contexts as well as we can or that participants are more likely to only take tests when they are at home. Due to the age of our cohort, most of our participants are retired and therefore may spend most of their time at home. Although overall adherence was high in this study, it is also possible that our participants chose not to complete tests when they were away from home, which may have led to an underestimation of the effects we observed. In other words, it might appear that older adults enrolled in EMA studies prefer home environments over completing tests “in the wild.” While EMA has a clear strength of being able to capture cognition (and other processes) in different environments, future studies should be aware that EMA studies of cognition in older adults might have limited sampling outside the home or in different social contexts because participants tend to complete assessments in minimally distracting and more controlled environments.

A second important implication of this work stems from our findings regarding the impact of environmental factors on cognition. Our participants frequently completed assessments in environments that could be considered distracting. Additionally, participants reported overt interruptions much more frequently than anticipated, during 12% of testing occasions. However, even after accounting for testing location, social context, and overt interruptions, the effects of environmental distractions had very little impact on performance for cognitively normal older adults. However, for those experiencing the earliest symptoms of dementia—in what could be considered the MCI stage, the effects of distractions were stronger, although still at a small effect size. EMA studies should routinely sample for location and social context and allow participants to report interruptions so that these can be included in statistical models. This is particularly important for studies that enroll participants with cognitive impairment.

These findings also have implications for understanding how distractions and interruptions impact functioning during everyday cognitively demanding tasks. Everyday tasks such as driving, managing money and medications, preparing meals, and general housekeeping require several different processes and are important for maintaining functional independence [35,36]. Being interrupted or distracted during these activities might lead to severe outcomes such as accidents or taking the wrong medicine. Our results suggest that techniques or interventions that teach individuals how to minimize distractions, especially during cognitively demanding tasks, might be useful to minimize adverse outcomes.

### Limitations and Future Directions

While the results of this study are intriguing and provide insight into the impact of environmental distractions on unsupervised cognitive assessments in older adults, it is worth noting some limitations. The sample used in this study consisted of older adults who are largely retired and therefore, may spend more time at home (which was reflected in the high proportion of tests completed at home, roughly 80%). This also likely contributed to the large standard errors for our estimates, especially among older adults with very mild dementia. Examining these effects in a cohort of younger participants, who are likely still employed and dealing with more day-to-day activities that require them to be out of the house, may more effectively examine the impacts of environmental distractions.

This study also relied on self-reports of testing location and social context, which may not capture objective levels of environmental distractions. Future research could consider incorporating continuous passive monitoring of environmental factors to better contextualize the effects. For example, GPS tracking could be used to detect differences in cognitive performance as a function of participants’ distance from home. Likewise, passive monitoring of noise levels from participants’ testing devices could be used to objectively quantify distractions in their environment. Additionally, our question regarding “interruptions” was not open-ended, and so we could not capture differences in the types of interruptions participants experienced. For example, notifications from other applications that popped up during the testing may differ in their distraction and impact on performance compared to interruptions from a family member calling for the participant. Future research should consider gathering more detailed information about the types of distractions or interruptions experienced by participants. Finally, this study focused only on the baseline assessment from ARC and how participants’ self-reported environmental factors were related to cognitive performance. Future work could investigate if there are longitudinal changes in participants’ environmental contexts (frequency of testing at home or alone) and how that is related to cognitive changes over time.

### Conclusions

Unsupervised EMA cognitive assessments are a unique data collection method that more closely represents how participants use cognition in their daily lives, but tests are often completed in distracting environments. Our results suggest that effects of environmental distractions, such as overt interruptions during testing and the location and social context in which tests are completed can have small impacts on cognition, and these effects were most apparent for older adults showing early symptoms of dementia. The effect sizes attributable to distractions in cognitively normal participants were very small and are not likely to threaten the validity or reliability of cognitive testing acquired in natural environments on mobile devices; thus, our results continue to support the validity of unsupervised ambulatory cognitive assessments.

## Supplementary material

10.2196/71578 Multimedia Appendix 1 Complete model outputs for linear effects models presented in main text.

## Abbreviations

AD: Alzheimer disease
ARC: Ambulatory Research in Cognition
CDR: Clinical Dementia Rating
CoV: coefficient of variation
EMA: ecological momentary assessment
Knight ADRC: Knight Alzheimer Disease Research Center
MCI: mild cognitive impairment
RT: reaction time
