# Supplementary material for: Impacts of Environmental Distractions and Interruptions on Unsupervised Digital Cognitive Assessments in Older Adults: Cognitive Ecological Momentary Assessment Study
Source: JMIR Mhealth Uhealth. 2025 Sep 8;13:e71578. doi: 10.2196/71578 (PMC12416871; doi:10.2196/71578)
Supplement: Multimedia Appendix 1 — Complete model outputs for linear effects models presented in main text. [file mhealth-v13-e71578-s001.docx]

**Table S1.** Full model output for mixed effect models the interaction between CDR status, testing location, social context on ARC performance (N=422).

|  | Prices Error Rate | Grids Euclidean Distance | Symbols Median RT | Symbols RT CoV |
| --- | --- | --- | --- | --- |
| **Predictors** | Estimate (SE) | Estimate  (SE) | Estimate  (SE) | Estimate (SE) |
| Baseline Age | 0.003 ^***^ (0.001) | 0.005 ^*^ (0.002) | 0.056 ^***^ (0.009) | 0.003 ^***^ (0.001) |
| Gender | 0.031 ^***^ (0.008) | –0.088 ^***^ (0.026) | 0.154  (0.104) | –0.004  (0.008) |
| Education | –0.006 ^***^ (0.002) | –0.017 ^**^ (0.005) | –0.007  (0.021) | 0.001  (0.002) |
| Sessions Completed | –0.000  (0.001) | –0.008 ^**^ (0.003) | –0.046 ^***^ (0.012) | –0.003 ^**^ (0.001) |
| CDR Status | 0.057 ^***^ (0.017) | 0.185 ^***^ (0.050) | 0.610 ^***^ (0.183) | 0.027  (0.015) |
| Home Prop | –0.015  (0.024) | 0.072  (0.072) | –0.131  (0.286) | –0.001  (0.022) |
| Alone Prop | 0.001  (0.014) | 0.020  (0.041) | –0.309  (0.163) | 0.016  (0.013) |
| Home | 0.004  (0.007) | 0.017  (0.019) | –0.032  (0.033) | 0.003  (0.006) |
| Alone | 0.006  (0.005) | 0.011  (0.012) | –0.022  (0.021) | 0.008 ^*^ (0.004) |
| CDR Status x Home | 0.046  (0.033) | –0.218 ^*^ (0.087) | –0.384 ^*^ (0.152) | 0.021  (0.027) |
| CDR Status x Alone | 0.004  (0.015) | 0.038  (0.040) | –0.049  (0.071) | 0.032 ^**^ (0.012) |
| Home x Alone | 0.024 ^**^ (0.009) | 0.048 ^*^ (0.024) | 0.070  (0.042) | 0.002  (0.008) |
| CDR Status x Home x Alone | –0.045  (0.039) | 0.260 ^*^ (0.102) | 0.401 ^*^ (0.178) | –0.015  (0.032) |

*Notes*. CDR = Clinical Dementia Rating. * *p* < .05, ** *p* < .01, *** *p* < .001.

**Table S2.** Full model output of mixed effect models the interaction between CDR status, testing location, social context on ARC performance after excluding sessions that were interrupted (N=422).

|  | Prices Error Rate | | Grids Euclidean Distance | | Symbols Median RT | | Symbols RT CoV | |  |
| --- | --- | --- | --- | --- | --- | --- | --- | --- | --- |
| **Predictors** | Estimate (SE) | | Estimate  (SE) | | Estimate  (SE) | | Estimate (SE) | |  |
| Baseline Age | | 0.003 ^***^ (0.001) | | 0.006 ^*^ (0.002) | | 0.055 ^***^ (0.008) | | 0.002 ^***^ (0.001) | |
| Gender | | 0.032 ^***^ (0.008) | | -0.087 ^***^ (0.026) | | 0.180  (0.097) | | 0.002  (0.007) | |
| Education | | -0.007 ^***^ (0.002) | | -0.019 ^***^ (0.005) | | -0.004  (0.020) | | 0.001  (0.001) | |
| Sessions Completed | | -0.001  (0.001) | | -0.008 ^**^ (0.003) | | -0.041 ^***^ (0.011) | | -0.001  (0.001) | |
| CDR Status | | 0.053 ^**^ (0.017) | | 0.198 ^***^ (0.050) | | 0.640 ^***^ (0.171) | | 0.030 ^*^ (0.014) | |
| Home Prop | | -0.015  (0.024) | | 0.051  (0.072) | | -0.087  (0.269) | | 0.001  (0.020) | |
| Alone Prop | | 0.005  (0.014) | | 0.019  (0.042) | | -0.286  (0.152) | | 0.022  (0.012) | |
| Home | | 0.003  (0.008) | | 0.009  (0.020) | | -0.062  (0.034) | | -0.000  (0.006) | |
| Alone | | 0.006  (0.005) | | 0.008  (0.013) | | -0.037  (0.022) | | 0.009 ^*^ (0.004) | |
| CDR Status x Home | | 0.055  (0.033) | | -0.243 ^**^ (0.086) | | -0.404 ^**^ (0.148) | | 0.025  (0.026) | |
| CDR Status x Alone | | 0.002  (0.016) | | 0.029  (0.041) | | -0.061  (0.072) | | 0.034 ^**^ (0.012) | |
| Home x Alone | | 0.022 ^*^ (0.010) | | 0.034  (0.026) | | 0.094 ^*^ (0.045) | | 0.001  (0.008) | |
| CDR Status x Home x Alone | | -0.057  (0.040) | | 0.287 ^**^ (0.102) | | 0.380 ^*^ (0.176) | | -0.030  (0.031) | |

*Notes*. CDR = Clinical Dementia Rating. * *p* < .05, ** *p* < .01, *** *p* < .001. Models adjusted for covariates including age, years of education, self-reported gender, and number of completed sessions. Full model output is provided in supplemental material. Home Prop = proportion of sessions completed at home. Alone Prop = proportion of sessions completed alone. Home = session completed at home (coded 0) or away from home (coded 1). Alone = session completed alone (coded 0) or in the presence of others (coded 1).
